# Can Nature Overcome Invasive Gastrointestinal Infections?

**DOI:** 10.3390/ijms26125795

**Published:** 2025-06-17

**Authors:** Anna Duda-Madej, Szymon Viscardi, Jakub Stecko, Natalia Szymańska, Ewa Topola, Katarzyna Pacyga, Marta Szandruk-Bender

**Affiliations:** 1 Department of Microbiology, Faculty of Medicine, Wroclaw Medical University, Chałubińskiego 4, 50-368 Wrocław, Poland; 2 Faculty of Medicine, Wroclaw Medical University, Ludwika Pasteura 1, 50-367 Wrocław, Poland; 3 Department of Environment Hygiene and Animal Welfare, Faculty of Biology and Animal Science, Wrocław University of Environmental and Life Sciences, 50-375 Wrocław, Poland; 4 Department of Pharmacology, Faculty of Medicine, Wroclaw Medical University, Mikulicza-Radeckiego 2, 50-345 Wrocław, Poland

**Keywords:** invasion, permeability, intestinal barrier, berberine, sanguinarine, cheleritrin

## Abstract

Invasive bacterial gastrointestinal infections represent a substantial clinical burden worldwide, contributing to significant morbidity and, in severe cases, mortality. The causative bacterial agents of these infections include *Shigella* spp., enteroinvasive *Escherichia coli*, *Salmonella* spp., *Campylobacter jejuni*, *Yersinia enterocolitica*, and *Listeria monocytogenes*. Given the growing challenges of therapy failures and rising antibiotic resistance, there is still an unmet need to identify novel, effective, and safe compounds exhibiting antimicrobial, anti-inflammatory, and immunomodulatory activities. In the present review, we aimed to compile current data regarding three alkaloids—berberine, sanguinarine, and cheleritrin—which hold significant promise in treating bacterial invasive gastrointestinal diseases. Our review extended beyond the direct antimicrobial properties of these compounds against pathogens capable of breaching the intestinal epithelial barrier. We also presented their modulatory effects on intestinal barrier integrity and their influence on the composition and function of the resident gut microbiota, thereby highlighting their potential indirect role in attenuating pathogen invasion and disease progression. Thus, our review presents alkaloids as potential preparations that potentiate the activity of classic anti-infective drugs, as well as substances that, by affecting the microbiome and intestinal mucosa, could be used for inflammatory bowel diseases.

## 1. Introduction

Bacterial, fungal, viral, or archaeal agents can cause gastrointestinal infections. They are a major health threat worldwide. According to data from the World Health Organization (WHO), an estimated 600 million people worldwide contract food-borne diseases each year, resulting in approximately 420,000 deaths [1]. Especially dangerous are infections with an etiology of microorganisms that show the ability to internalize. In this case, the main sources of infection include (i) direct contact with an infected person (*Shigella* spp.), (ii) ingestion of contaminated water or food (*Shigella* spp., enteroinvasive *Escherichia coli* (EIEC), and *Listeria monocytogenes*), (iii) consumption of raw or undercooked poultry or pork (non-typhoidal *Salmonella* spp. (NTS), *Campylobacter jejuni*, and *Yersinia enterocolitica*), (iv) consumption of unpasteurized milk and dairy products (NTS, *Y. enterocolitica*, and *C. jejuni*), and (v) contact with domestic animals (*C. jejuni*).

The bacteria responsible for these infections not only produce virulence factors—such as cytotoxins and invasins—that facilitate entry into intestinal epithelial cells but also possess the ability to penetrate the intestinal mucosa, leading to tissue damage and, in some cases, erythrocyte destruction. Pathogens exhibiting these capabilities include *Shigella* spp., *Salmonella* spp., *C. jejuni*, *L. monocytogenes*, and *Y. enterocolitica*, as well as EIEC [2,3,4,5,6]. Their pathogenic mechanisms aim to (i) breach the intestinal barrier, (ii) survive and replicate within the intestinal lumen or inside macrophages, and (iii) induce tissue injury and inflammatory responses [7].

**Figure 1:**
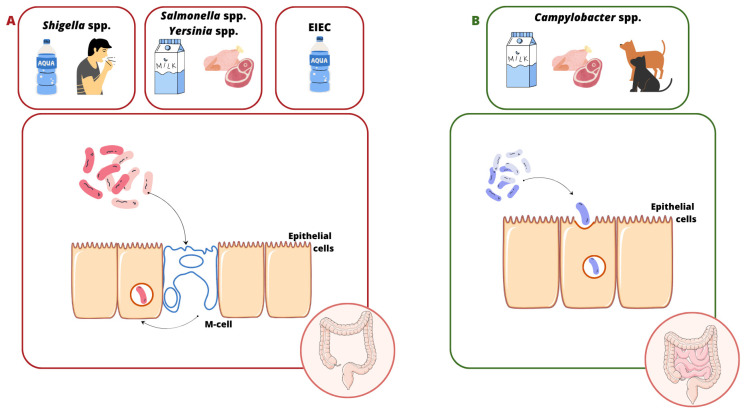
Source and transmission of infections caused by invasive pathogens. (**A**) The *Shigella* spp., *Salmonella* spp., EIEC, and *Yersinia enterocolitica* internalization process. These bacteria penetrate M cells in Peyer’s tufts, leading to colon damage. (**B**) *Campylobacter jejuni*’s invasion into host cells. This takes place by endocytosis, leading to damage to the ileum and colon.

**Figure 2:**
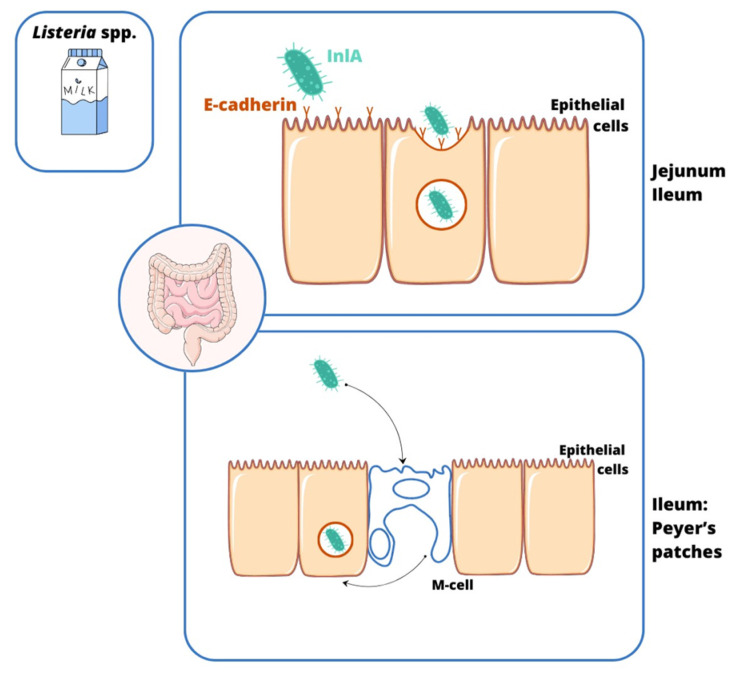
The source and transmission of *Listeria monocytogenes* infections. The specialized process of internalization of *L. monocytogenes* into the epithelium of the gastrointestinal tract is carried out by (1) the transcytosis of M cells in Peyer’s clusters and (2) the binding of internalin A (InlA) to E-cadherin on enterocyte microvilli, leading to damage to the ileum and colon.

The invasion process is a complex process involving a specific interaction between the microorganism and the host cell. Invasive bacteria can enter colonic epithelial cells through M cells in Peyer’s tufts (*Shigella* spp., *Salmonella* spp., EIEC, *L. monocytogenes*, and *Y. enterocolitica*). Afterwards, some of them adapt to the conditions and proliferate intracellularly (e.g., *Salmonella* spp., EIEC, and *Y. enterocolitica*), while others leave the phagosome and enter the cytoplasm (e.g., *Shigella* spp.) [7]. The majority of these bacteria show the ability to mechanically damage the mucosa, causing inflammation (e.g., *Shigella* spp., EIEC, *Y. enterocolitica*, and *Salmonella* spp.) [8], but some of them can also enter the blood, leading to systemic infection (e.g., *Salmonella enterica* serovar Typhi and *L. monocytogenes*) [9,10]. A slightly different infection mechanism is observed in infections caused by *C. jejuni*. This pathogen enters the intestinal cells by endocytosis (only some strains penetrate M cells). This process depends on (i) the host, (ii) the reorganization of the actin cytoskeleton, and (iii) the production of invasive proteins, i.e., Campylobacter adhesion to fibronectin (CadF), fibronectin-like protein A (FlpA) [11], and Campylobacter invasive antigens (Cia) [12]. The cell cycle shutdown at the G2 stage occurs with the involvement of CDT (cytolethal distending toxin), which damages the DNA of the enterocyte, leading to its swelling and apoptosis [13,14] (Figure 1). Also, the way *L. monocytogenes* penetrates the gastrointestinal tract differentiates it from the invasive pathogens mentioned here. For this purpose, the bacterium uses not only M cells, but especially the produced internalin A (InlA), which binds to E-cadherin located on the peaks of enterocytes. Its site of invasion is the small intestine, the ileum and jejunum (Figure 2). This infection pattern explains why *L. monocytogenes* mainly infects humans rather than other species. This is due to the easy accessibility of this protein on the cell surface [10,15]. A point worth noting is that infections with strains with invasive potential not only manifest as acute bloody diarrhea, but can also lead to serious complications, i.e., (1) *C. jejuni*: Guillain–Barré syndrome, reactive arthritis, and systemic infections [16,17,18,19,20]; (2) *Shigella* spp.: hemolytic uremic syndrome (HUS) [21,22]; (3) *S.* Typhi: meningitis, encephalitis, peritonitis, and delirium [23,24,25]; (4) *Y. enterocolitica*: Reiter’s syndrome, reactive arthritis, erythema nodosum, myocarditis, and glomerulonephritis [26,27]; and (5) *L. monocytogenes*: sepsis, encephalitis, meningitis, miscarriage, and stillbirth [28].

The problem of invasive bacterial gastrointestinal infections is not only a clinical challenge, but also a significant public health threat that requires a comprehensive approach at the global level—from animal control to proper food hygiene. In this review, we aimed to collect data on the three alkaloids—BBR, CHE, and SAN—with the best promise, based on the available literature, in controlling invasive bacterial gastrointestinal diseases. We analyzed the problem not only from the side of antimicrobial activity of these compounds against pathogens, showing the ability to penetrate the intestinal epithelial cell, but also by looking at and analyzing the existing problem from the side of the effect of these alkaloids on the permeability of the intestinal barrier and their effect on the colonizing microbiota, and thus demonstrated their indirect involvement in the invasion process.

## 2. Molecular Basis of Enteroinvasive Pathogen Virulence

The pathogens capable of causing enteroinvasive infections described in this review have developed a variety of membrane molecules by which they induce colonization and then internalization into enterocytes. The internalization process is mediated by numerous virulence factors, including secretion systems (including T3SS) by which bacteria introduce other virulence factors into cells. The introduced molecules inhibit the fusion of phagosomes with lysosomes, protect bacterial cells against oxidative stress, modify signaling pathways, or remodel the actin cytoskeleton, which allows proper invasion by, among others, forming endosomes. Specific invasive pathogens, e.g., *Shigella* spp. and *L. monocytogenes*, present a unique way to infiltrate neighboring cells through the F-actin polymerization of the so-called actin tail that is formed. After infiltration of the mucous membrane, it is possible to further spread the bacteria to the body, which, however, requires other mechanisms of pathogenicity from the bacteria, e.g., intensification of the inflammatory response, which results in tissue damage; induction of phagocyte apoptosis; and phagocytosis and secondary spread to lymphatic structures.

In the following section, we will present the molecular aspect of infections caused by several invasive pathogens: *Shigella* spp., *Salmonella* spp., *Campylobacter* spp., *Yersinia* spp., *L. monocytogenes*, and EIEC.

For the *Shigella* non-dysenteric genus, e.g., *Shigella flexneri*, *sonnei*, and *boydi* as well as *dysenteriae*, the invasion of intestinal cells begins in the area of the colon where the bacterium performs glycosylation of protective mucins, leading to the formation of a gel in which it then vegetates [29,30]. This is followed by the type III (T3SS), type VI (T6SS *S. sonnei*), and type II (T2SS *S. boydi*) secretion systems—key factors in invasion propagation [30,31,32,33]. After the initial internalization of *Shigella* cells by M cells of Peyer patches, they are transported via transcytosis and exposed to cells located deeper (*lamina propria*), e.g., macrophages [30]. M cells are specialized cells of the intestinal mucosa that perform numerous immune functions, including absorbing foreign antigens and then, by transcytosis, transferring them to cells located below that are capable of presenting antigens (APCs), e.g., dendritic cells. [34]. The described mechanism allows for the transport of antigens from the intestinal lumen to MALT (mucosa-associated lymphoid tissue) immune cells and enables the generation of an immune response to, among others, infections [34]. Due to the above-mentioned process of non-specific immunity, invasive pathogens are able to penetrate the epithelial barrier and infiltrate the *lamina propria* of the mucous membrane. After the induction of macrophage apoptosis, *Shigella*, via T3SS (depending on the presence of the pINV plasmid), introduces protein effectors into enterocytes [35]. Through the initial interaction of the outer membrane protein IcsA (encoded by the *Vir*G gene) with the host cell, membrane pore formation is initiated by the bacterial proteins IpaB, IpaC, and IpaD [31,36]. Through the resulting T3SS needle, virulence factors (VirA, IpaA-C, and IPgD) enter the host cell, which results in the process of disrupting enterocyte signaling pathways and regrouping the cytoskeleton (depolymerization of actin, formation of endocytic vacuole, and phagosome escape). Activation of the N-WASP (neuronal Wiskott–Aldrich syndrome protein) due to these factors leads to the activation of Arp2/3 complexes, which finally reorganize actin fibers, inducing endocytosis in the interior of the cells [31,37]. Another group of T3SS effectors, IpgB1 and IpgB2 proteins, by interaction with GTPases of the Rho type, stimulate their activation and consequently, among others, ruffling of the enterocyte cell membrane [38,39,40]. After internalization of the phagosome, the bacterium avoids being digested by lysosomes and by, for example, the IpaB factor—an inducer of escape from the phagosome to the cytosol [41]. The next step is the polymerization of actin fibers at the pole of the bacterial cell via the IcsA protein, thus forming a comet tail, as in the case of *Listeria*, which allows the invasion of neighboring cells and migration of the pathogen [42,43]. Bacteria living in the cytoplasm of enterocytes induce NOD (nucleotide-binding oligomerization domain-like receptors), resulting in the activation of the NF-κB signaling pathway and, finally, the secretion of the neutrophil chemoattractant IL-8 [44]. The migration of neutrophils and the degradation of tight junctions between enterocytes cause the mucosa to leak and facilitate further penetration of the bacteria [45]. In the case of *S. dysenteriae* type 1, after initial adhesion identical to that present in non-dysenteric strains, the bacterium can introduce protein effectors into enterocytes via the T2SS system, but virulence focuses mainly on the secretion of Shiga toxin (Stx) [32,46].

In a similar way to *Shigella* spp., the process of the invasion of intestinal cells by EIEC takes place. The pathogen in phylogenetic terms is so closely related to *Shigella* that some researchers postulate its inclusion in the genus *Escherichia* [47]. The significant similarity between *Shigella* and EIEC is largely due to the fact that both pathogens carry the pINV invasiveness plasmid, which encodes the protein effectors of invasion and virulence [48]. Similarly to *Shigella* spp., the EIEC exhibits M-cell-targeted tropism when it penetrates the described cells, and then the bacterium undergoes transcytosis into the underlying macrophages [49]. Bacteria leave apoptotic or pyroptotic macrophages and invade enterocytes using, e.g., T3SS. IpaB-D effectors and IpaA-C, VirA, and IPgD virulence factors stimulate actin cytoskeleton rearrangement, which, in the long term, causes membrane ruffling and endocytosis [47,50]. The escape from the phagosome to the cytoplasm is associated with the same virulence factors (e.g., IpaB), followed by actin tail formation [47,51]. The described mechanism potentiates the infection of subsequent intestinal cells; however, it is worth noting that the process itself proceeds with a lower efficiency than in *Shigella* spp. [52].

**Figure 3:**
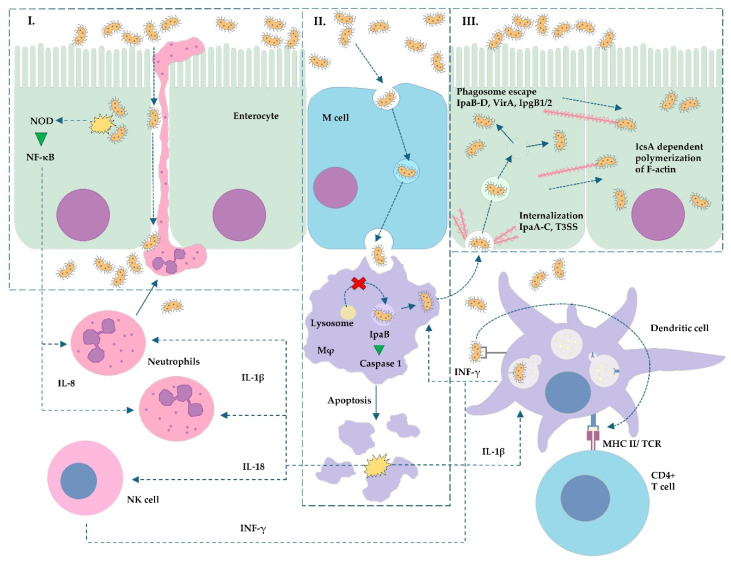
Simplified scheme of *Shigella* spp. and EIEC enteroinvasion: (**I**) Bacterial internalization causing NOD activation and NF-κB signaling pathway upregulation resulting in IL-8 secretion. Neutrophil migration and activation cause the degradation of tight junctions (occludins and claudins), which potentiate bacterial infiltration of submucosal tissue. (**II**) Internalization of bacterial cells into M cells and their transcytosis to macrophages located downstream. Fusion of lysosome with endosome is inhibited, the bacterium escapes from the endosome to the cytosol, and virulence factor IpaB promotes apoptosis of macrophages. (**III**) *Shigella*/EIEC, after escaping from the macrophage, invades the enterocyte via T3SS and its effectors (e.g., IpaA-C). Then, the bacterium induces phagosome lysis and translocates into the cytosol, and the IcsA protein induces the formation of F-actin polymer (actin tail). The pathogen migrates to the surrounding enterocytes. Elements common to other pathogens can be found in the scheme: *L. monocytogenes* ((**III**) formation of actin tail), *Salmonella* and *Yersinia* ((**I**–**III**) the difference is that these pathogens do not polymerize F-actin and stay in primary formed vacuoles inside phagocytes or enterocytes—lack of phagosome escape). Abbreviations: CD4—cluster of differentiation 4; IcsA—Intracellular chromosome-mediated spreading Actin-based motility protein; IL—interleukin; INF-γ—interferon gamma; IpaA-D, IpgB1/2—invasiveness factors of Shigella/EIEC; Mφ—macrophage; MHC II—Major Histocompatibility Complex class II; NF-κB—nuclear factor κB; NK cell—natural killer type cell; NOD—nucleotide-binding oligomerization domain-like receptors; TCR—T-cell receptor; T3SS—type 3 secretory system; VirA—Virulence-associated protein A.

The summary of the *Shigella* spp. and EIEC invasion process is depicted in Figure 3.

*Salmonella enterica* includes more than 2500 serotypes collected in serovars, of which invasive infections are most often caused by *S.* Typhi and less often by infection with serovars Typhimurium (*S.* Typhimurium), Enteritidis (*S*. Enteritidis), and Choleraesuis (*S*. Choleraesuis) [53,54]. Like *Shigella* spp. and *Yersinia* spp., *Salmonella enterica* presents tropism to M cells located in the distal ileum, which facilitates its penetration through the intestinal barrier [55]. The potential for invasive infections is due to the presence of virulence factors grouped in areas of the genome called pathogenicity islands (SPI—*Salmonella* pathogenicity island), of which SPI-1 and -2 are key in invasion [56,57]. Penetration into M cells is the result of the interaction of TLRs (toll-like receptors) with *Salmonella* surface antigens. After phagocytosis, bacteria are transferred to the *lamina propria* of the mucous membrane. Bacteria undergo phagocytosis by macrophages, as in the case of *Shigella*, where they induce their apoptosis and then invade enterocytes, as well as other phagocytes or dendritic cells [58]. Thanks to T3SS encoded by SPI-1, the pathogen introduces virulence factors into the host cell, including SopA-E and SipA-B (actin remodeling, tight junction degradation, and phagocyte apoptosis induction) [59,60]. This results in membrane ruffling and subsequent bacterial internalization in *Salmonella*-containing vacuoles (SCVs) [61]. Bacteria can be altered by the expression of SPI-2 effectors, e.g., SpiC inhibits SCV fusion with lysosome and blocks the MHC-II expression of dendritic cells, while SseB, SsrA, SsaJ, and Ssav inhibit oxidative burst [62,63,64]. Importantly, through other virulence factors, the pathogen can induce both the apoptosis and pyroptosis of phagocytic cells, which enables further spread of invasion [65,66].

The *Yersiniae* genus contains two significantly pathogenic species causing invasive infections in humans: *Y. enterocolitica* and *pseudotuberculosis*. The ability to invade enterocytes is due to the presence in these pathogens of the specific plasmid pYV, which encodes important virulence factors [67,68]. Due to OMPs (outer membrane proteins), bacteria are internalized in M cells of Peyer’s patches in the terminal area of the ileum, where the key role is played by specific invasins and the Ail protein [69,70]. Like other *Enterobacteriaceae*, *Yersinia* forms T3SS, but unlike *Salmonella*, its effectors (YopH, E, M, O, P, and T) mainly inhibit phagocytosis and modulate inflammatory pathways to promote intracellular survival [71]. These proteins enable the *Yersinia* cells to move to the basolateral side of the intestinal cells, and invasins are connected to the β1-integrins. [72]. This induces the remodeling of the actin cytoskeleton and the internalization of bacteria into epithelial cells [73]. The main bacterial virulence factor—the YadA protein (expressed in Peyer patches)—then allows adhesion to collagen types I-V and XI [74], fibronectin, and laminin 1 and 2, resulting in adhesion to epithelium and immune cells [75]. The described interaction leads to the formation of YadA-ECM (extracellular matrix) complexes, resulting in the induction of β1-integrin-dependent signal cascades, which promote actin remodeling and the secretion of pro-inflammatory cytokines, e.g., IL-8 [76]. Because YadA provides protection against phagocytosis, inflammation does not affect bacteria, but damages the mucous membrane, which facilitates further invasion [77,78].

*Campylobacter* spp. interact with intestinal cells via outer membrane proteins (OMPs), of which the most important molecules are CadF (fibronectin-binding protein), FlpA (fibronectin-like protein A), and JlpA (jejuni lipoprotein A-binding protein Hsp90) [79,80,81]. The adhesion properties of the HtrA protein, which is associated with the process of the degradation of the connections between enterocytes, such as occludins and claudins, have also been described [82]. Further invasion is a consequence of paracellular cell migration to the basolateral side of the enterocyte, and paving the way is made possible by modifying the fibronectin molecules and degrading the tight junctions (occludins and claudins) by the HtrA protease [83,84]. Subsequently, the interaction of CadF and FlpA with fibronectin from the basolateral side of enterocytes induces the remodeling of the actin cytoskeleton (mediated by integrin signaling) and ruffling of the cell membrane [85]. Then, the activated intercellular signaling pathway leads to the reorganization of actin filaments—ruffling of the cell membrane and the endocytosis of *Campylobacter* [85,86,87]. The bacterium transferred into enterocytes forms the so-called CCV (*Campylobacter*-containing vacuole), which avoids fusion with the lysosome by interference with EEA-1 (early endosomal agent 1) and GTPases Rab4/5 [88,89]. The result is the intracellular survival of bacteria, their division, and further invasion.

Different surface ligands of enterocytes are associated with the *L. monocytogenes* invasion process [15]. The pathogen invades host cells by specific internalins: InlA- and InlB-binding proteins, such as E-cadherin and hepatocyte growth factor receptor Met, respectively [90,91,92]. Some other OMPs play an important role in the adhesion process, e.g., InlE, InlG, and InlH [93]. After initial adhesion, the interaction between the InlA and E-cadherin molecules triggers a caveolin-dependent grouping of bound E-cadherins in clathrin-rich membrane regions [94,95]. This can further induce caveolin or clathrin-dependent endocytosis, which allows for proper invasion [96,97]. After entering the cell in the form of an endosome, the bacterium, due to, among others, listeriolysin O (LLO), degrades the vacuole membrane and penetrates into the cytosol of an infected cell [98,99]. Other potential triggers of *Listeria* escape from the phagosome have also been described: GILT (gamma-interferon-inducible lysosomal thiol reductase), Rab5a GTPase, and pPplA (peptide pheromone-encoding lipoprotein A) [100,101,102]. An important virulence factor involved in bacteria migration between infected cells is ActA, thanks to which the polymerization of host F-actin occurs at the pole of the *Listeria* cell. The formation of actin comet tails is recognized as a key motor force of the pathogen in the process of the invasion of neighboring cells [103,104]. The spread of bacteria in the body occurs after bacteria cross the *lamina propria* of the mucous membrane; hence, *Listeria* migrate via the bloodstream to the parenteral areas, e.g., the liver, placenta, or central nervous system [105].

An additional problem in controlling invasive infections is antibiotic resistance. Strains resistant to fluoroquinolones and macrolides—the main groups of antibiotics used to treat severe cases of yersiniosis, campylobacteriosis, or typhoid fever—are increasingly being isolated. This phenomenon is largely attributed to the widespread use of these antimicrobials in animal husbandry, which contributes to the selection and dissemination of resistant strains [106]. Therefore, there is a need to look for new active compounds with antimicrobial, anti-inflammatory, and immunomodulatory properties, which may provide a valuable complement to the treatment of invasive gastrointestinal infections. Many of the recently studied compounds with antimicrobial, anti-inflammatory, and immunomodulatory effects are of plant origin. It is important that these compounds not only support antibiotic therapy but also effectively enhance the intestinal barrier. A group of compounds with proven activity in gastrointestinal infections, including invasive infections, is alkaloids. Of these, berberine (BBR), sanguinarine (SAN), and cheleritrin (CHE) are particularly noteworthy. Of these, BBR is the best-documented compound in the literature. This alkaloid has a proven ability to inhibit invasion into intestinal epithelial cells [107], reduce inflammation (affecting the production of pro-inflammatory cytokines, i.e., IL-1β, TNF-α, and IL-6) [108,109], and protect the tight junctions between intestinal cells [110,111,112,113]. CHE, on the other hand, has a confirmed strong inhibitory effect on bacterial adhesion to the epithelium [114,115,116]. Furthermore, it has been shown to have a protective effect on the intestines by accelerating their recovery from previous damage [117]. SAN and its derivatives also exhibit potent anti-inflammatory activity, attractive activity against intestinal pathogens, and inhibit invasion ability [9,118,119]. However, considering the high cytotoxicity of CHE and SAN, their helpful participation in gastrointestinal infections must take into account the need to modify the form of their administration (e.g., using carriers, i.e., nanoparticles).

## 3. Berberine

### 3.1. Characteristics

**Figure 4:**
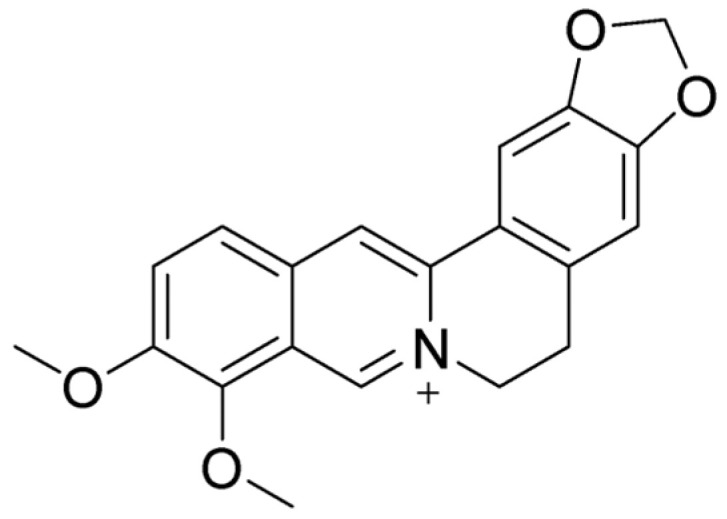
Molecular structure of BBR.

Berberine (BBR) (Figure 4) is a naturally occurring isoquinoline alkaloid commonly found in medicinal plants such as those from the *Berberis*, *Coptis*, and *Hydrastis* genera. Known for its bright yellow color, berberine is celebrated for its wide range of pharmacological properties [108,120]. These include antidiabetic [121], anticancer [122], anti-inflammatory [123], antimicrobial [124], hypolipidemic [125], and neuroprotective effects [126]. Despite its potential, its clinical use is hindered by poor solubility and low bioavailability [127]. Historically, BBR has been used to treat diarrhea and gastroenteritis, showing effectiveness against bacterial infections in the gastrointestinal (GI) tract [128]. It has also been found to alleviate symptoms of irritable bowel syndrome (IBS), particularly the diarrhea-predominant subtype (IBS-D), by targeting opioid receptors and reducing visceral pain and excessive bowel movements [129]. Moreover, BBR has demonstrated the ability to repair colonic damage and modulate gut microbiota, highlighting its potential as a treatment for inflammatory bowel disease (IBD) [130]. These findings suggest that BBR holds significant promise as a versatile therapeutic agent, though further research is needed to overcome its bioavailability challenges.

### 3.2. Impact on Intestinal Mucosa

In addition to the above-mentioned properties of alkaloids, BBR has an impact on intestinal cells, including the enteric nervous system (ENS), intestinal epithelial cells (IECs), intestinal stem cells (ISCs), and intestinal mucosal immune cells [131,132,133]. Enteric glial cells (EGCs) are a major component of the ENS and play an important role in the regulation of intestinal inflammation [132]. EGCs are able to receive and respond to signals through the expression of functional pattern recognition receptors, cytokine receptors, nuclear factor kappa of activated B cells (NF-κB), MYD88, and signal transducers and activators of transcription (STATs) [134]. Furthermore, EGCs secrete cytokines, chemokines, and neuropeptides like glial cell-derived neurotrophic factors (GDNFs), substance P, neurotrophins, and calcitonin gene-related peptides (CGRPs) to regulate immune responses, including chemotaxis, lymphocyte mitogenesis, phagocytosis, and immune cell homing patterns [132,135].

BBR adjusts the interaction between EGCs, IECs, and immune cells and ameliorates intestinal inflammation [132,136]. The downregulation of adhesive molecules and the inhibition of chemokine secretion were observed, which resulted in the suppression of adhesion and chemotaxis of immune cells toward IECs [132]. Ultimately, BBR lowers intestinal permeability and reduces inflammatory infiltration [132].

The study on Caco-2 cell monolayers demonstrated that berberine can reconstruct gut barrier integrity [137]. Treatment with BBR can restore the expression of tight junction proteins such as ZO-1, claudin-2, and occludin [137]. However, this ability of BBR is indissolubly connected with the intestinal microbiome. Through the regulation of the gut microbiota-associated tryptophan metabolite, BBR activates the aryl hydrocarbon receptor (AhR), which can greatly improve the disrupted gut barrier function [137]. ISCs are the only dividing cells in the intestinal epithelium and enable its regeneration. As it is a highly sensitive tissue, infections and inflammations can easily damage epithelial cells. BBR significantly enhanced the proliferation and differentiation of ISCs by modulating the activity of the mTORC1, STAT3, and ERK1/2 signaling pathways in both healthy and injured mice [133].

### 3.3. Impact on Gut Microbiome and Its Clinical Implications

One of the most prominent effects of BBR on the gastrointestinal tract is its modulatory impact on the intestinal microbiota. Due to its poor bioavailability after oral administration, berberine interacts directly with gut bacteria in the intestinal lumen [131,138]. Studies on various animal models afflicted with different diseases showed that BBR can regulate gut microbiota and reestablish an intestinal homeostasis that was disturbed as a result of the development of these disorders [130,131,137,139,140]. 

In ulcerative colitis (UC) in rats and mice, the BBR administration decreased the amount of conditional pathogenic bacteria like *Mucispirillum*, *Oscillospira*, *Bacteroides uniformis*, *Allobaculum* [130], and other harmful bacteria such as mouse intestinal *Bacteroides*, segmented filamentous bacteria, and *Enterobacteriaceae* [131], as well as increased the level of lactic acid-producing bacteria [130], *Bacteroides fragilis* [140], *Lactobacillus*, *Lactococcus*, and *Akkermansia*, which can act as anti-inflammatory agents and restore gut barrier function [137]. Similar results have been derived from studies on BBR treatment in type 2 diabetic mice and rats [141,142,143,144,145]. Significant growth of *Faecalibacterium*, *Roseburia*, *Clostridium* XIVa, *Ruminococcus2*, *Dorea*, *Butyricicoccus*, and *Coprococcus* was observed. These bacteria are widely known butyrate producers that can act as probiotics and exert a positive influence on the intestine [143]. Butyrate is a short-chain fatty acid and plays a pivotal role in maintaining a healthy intestine [146]. It acts as a primary energy source for colonocytes (the cells lining the colon) and exerts a range of beneficial effects crucial for gut integrity and overall well-being [147]. Moreover, butyrate exhibits anti-inflammatory effects through the modulation of the activity of the immune cells and enhances gut barrier function by promoting the production of mucus and regulating tight junction proteins [148]. On the other hand, BBR, both with and without stachyose, lowers the amount of *Enterobacteriaceae* and *Proteobacteria*, but also beneficial species such as *Bifidobacteriace* [141]. As BBR has a great influence on gut microbiota, its properties were under investigation in the mouse model for anti-diarrhea properties. Although BBR was not able to control *Clostridioides difficile* infections in monotherapy, it was effective in combination with antibiotics [149,150]. Conventional antibiotic therapy inhibits the growth of *C. difficile* and other *Firmicutes* but disturbs the balance and homeostasis in gut microbiota. As BBR can restore intestinal microbiota, it seems to be a good supplementation for an antibiotic treatment [149,150]. What is more, BBR plays an inhibiting role on gastrointestinal (GI) mobility in rodents through downregulating motilin and gastrin and upregulating somatostatin and glucagon-like-peptide-1 (Glp-1) [151]. BBR-induced modulation of microbiota improves metabolic disorders, including the previously mentioned diabetes and hyperlipidemia [152]. Furthermore, numerous other studies on animal models, concerning mice and rats with colorectal cancer, atherosclerosis, liver diseases, and neurological, psychiatric, and autoimmune disorders, demonstrated the importance of intestinal microbiota changes [139,153,154,155,156,157,158]. Since the results of animal studies look promising, there have also been clinical trials on patients. According to ClinicalTrials.gov, there are 58 completed studies: 20 that are still looking for participants; 3 that are active, but not recruiting; 4 that were withdrawn; and 20 with an unknown status. Out of them, 18 concern gastrointestinal diseases generally, and 4 are focused on gut microbiota. A randomized, double-blind, placebo-controlled trial with newly diagnosed type 2 diabetes patients demonstrated that BBR decreased the level of the species that mainly produce single sugar or SCFAs from fermenting polysaccharides or oligosaccharides, including *Roseburia* spp., *Ruminococcus bromii*, *Faecalibacterium prausnitzii*, and *Bifidobacterium* spp., and increased the amount of two *Bacteroides* spp. and multiple taxa of γ-*Proteobacteria* [128]. Changes in *Bifidobacterium* spp. are worthy of particular attention as these are beneficial species, and the supplementation of probiotics should be considered, especially in older diabetic patients [128]. Notably, the 18-week, multi-center, randomized, double-blind, parallel-controlled study of patients newly diagnosed with hyperglycemia showed the potential of *Bifidobacterium* spp. to enhance BBR’s hypoglycemic effects [159]. However, BBR dosage differed in both studies [128,159]. The conflicting information regarding *Bifidobacterium* spp. (decreased by BBR in one study, yet enhanced its effect in another) underscores the complexity of host–microbe–drug interactions and highlights the critical importance of dosage. Future research should focus on dose optimization to maximize therapeutic benefits while minimizing detrimental microbial shifts, investigating the potential of co-therapies (e.g., specific probiotics or prebiotics) to mitigate negative microbial changes and enhance positive ones. Long-term studies are necessary to assess the overall safety and efficacy profile considering these microbiome interactions. The impact of BBR on the human gut microbiota is summarized in Table 1.

### 3.4. Antimicrobial Properties Against Various Enteroinvasive Bacterial Pathogens

*Listeria monocytogenes*

BBR showed additive pharmacological activity with aminoglycoside–streptomycin (STM). Alkaloids obtained a MIC = 8192 µg/mL (MIC for STM = 8 µg/mL) against *L. monocytogenes* CMCC 54004 [160]. Li et al. investigated the photochemical antibacterial properties of BBR against *L. monocytogenes* ATCC 13932. Researchers have shown that BBR can generate ROS through a photodynamic reaction. While the alkaloid in the dark did not cause a significant antimicrobial effect even at a high concentration of 100 μM, under the influence of light, it led to a total eradication (a decrease of 10^5^ CFU/mL) of this strain at a concentration of half the initial value. Similar observations are derived from the analysis of the antibiofilm activity of the alkaloid at a concentration of 100 µM (eradication of mature biofilm with photodynamic reaction at the level of 63.6% vs. in the dark at 22.8%). At the molecular level, it was shown that the reaction led to the inhibition of β-galactosidase and interference with DNA (impaired expression of genes: hemolysin (*hly*), actin-inducing protein (*act*A), and 16SrRNA) [161]. Kim et al. investigated the activity of N-Mannich bases of BBR linking piperazine against *L. monocytogenes*. It was noted that these derivatives at a concentration of 200 µg/mL reduced the ability to grow a mature biofilm by >50%. Microscopic analyses showed that exposure to derivatives led to impaired cell density and aggregation in the biofilm area [162].

*Salmonella* spp.

Cheng et al. investigated the antimicrobial properties of BBR and its derivatives, including tetrahydroprotoberberine (THPBBR) and N-methyl-THPBBR. The compounds were characterized by different degrees of activity against *S.* Choleraesuis CVCC 504. In the Kirby–Bauer analysis, BBR obtained a ZOI diameter of 24 mm, which made it the most active compound of all the compounds tested (THPBBR: 7 mm; N-methyl-THPBBR: 6.4–7 mm). The microdilution test confirmed these reports: MIC for BBR of 62.5 μg/mL vs. other derivatives at 250–500 μg/mL. [163]. Moreover, pharmacological interactions between BBR, ciprofloxacin, and CYP (an antibiotic often used in the treatment of *Salmonella* spp. infections), as well as the ability of BBR to interfere with the formation of biofilms by *S. enterica* subsp. *enterica* serovar Gallinarum, were also investigated. Among the tested strains (CVCC 528 and MDR strain), the MIC for BBR was shown at the level of 3.125 μg/mL, and the checkboard analysis showed a synergistic relationship between the alkaloid and CYP. Importantly, it was shown that in subinhibitory concentrations, both substances (½ and ¼ MIC) significantly reduced the biofilm adhesion rate of *S.* Choleraesuis, as well as inhibited the further growth of biofilms. SEM analysis showed a number of molecular changes at the cellular level, including a decrease in the thickness and dimensions of the biofilm layer. The detection of inhibition of the expression of key genes/virulence factors responsible for the formation of the biofilm structure, including *lux*S, *rpo*E, and *omp*R, was also carried out [164]. BBR was also revealed to have its potential pharmacological interactions with the aminoglycoside STM. Alkaloids were weakly active against *S.* Typhimurium SL1344, MIC = 2048 µg/mL (STM MIC = 4 µg/mL); however, it was noted that in combination, compounds showed an additive relationship [160]. Jeyakkumar et al. described the novel BBR–benzimidazole derivatives’ activity against *S.* Typhi. The three groups of derivatives were tested for antibacterial properties and obtained the following MIC values: 16–64 μg/mL, 2–512 µg/mL, and 2–256 μg/mL. The antibiotics used in the controls showed comparable or weaker effects than the tested preparations (MIC for chloromycin equal to 32 µg/mL and norfloxacin equal to 4 μg/mL). Moreover, interestingly, it has been shown that one of the derivatives was characterized by an increased ability to perforate the cell membrane of Gram (-) bacteria as well as the fragmentation of DNA material [165]. The properties of BBR in the field of the inhibition of adhesion and the invasiveness of *S.* Typhimurium were also examined. It was shown that the alkaloid was significantly active against the pathogen; the MIC value was set at 0.076 mg/mL. It was also proven that the preparation was characterized by a strong antibiofilm activity already at a subinhibitory concentration of 0.019 mg/mL (inhibition rate of 31.20%). Importantly, in an in vivo model of the etiology of *Caenorhabditis elegans Salmonella* infection, BBR therapy was shown to significantly reduce the nematodes’ paralysis rate (at a concentration of 0.038 mg/mL, a decrease from 85% to 65.38%) [166].

BBR has also been shown to potentiate the action against the AcrAB-TolC efflux pump of *S.* Typhimurium MDR strains of various plant extracts. Particularly preferred was the relationship between alkaloids and methanol extracts of *Zingiber officinale*, in the case of which a reduction in the MIC of BBR of 4-fold (32–64 µg/mL) was observed. The observed increased accumulation of ethidium bromide in *Salmonella* cells exposed to BBR proved the existence of the mechanism of action of the alkaloid, which may lead to disruptions in the efflux and allow for overcoming this type of resistance [167,168]. Xu et al. examined the effect of BBR on the expression of genes encoding type I fimbriae in *S.* Typhimurium (*fim*A, *fim*H) and the accompanying implications for the pathogen’s ability to form biofilms. MIC values for individual strains were as follows: wild type and *fim*H mutant strain 0.9 mg/mL; *fim*A mutant strain 1.0 mg/mL. A subinhibitory concentration of 1/16 MIC was shown to lead to a significant decrease in type I fimbriae formation compared to the untreated control (SEM analysis). Interestingly, it has been shown that with the increase in BBR concentration, the expression of the *fim*A gene decreases and *fim*H increases. The described molecular effects led to the impairment of the ability of bacteria to aggregate and form biofilms (for the wild type, a decrease in biofilm formation by 66.29% at 1/16 of MIC for BBR) [169]. Naz et al. have shown that BBR can interfere with the cell division protein FtsZ in *S.* Typhi, and it also exhibits bactericidal activity against several bacterial pathogens. It has been observed that the substance led to the inhibition of the polymerization (inhibition rate ~70%) of the FtsZ protein and consequently disrupted cell division (in silico). The MIC for *Salmonella* was estimated at 500 μg/mL [170]. BBR also showed moderate activity against *S.* Typhimurium ATCC 14028. In view of the mentioned pathogen, the MIC value was set at 512 μg/mL, which is interesting because the preparation was therefore as active against the bacteria as vancomycin (VAN) [171]. Haque et al. reported on the unique molecular mechanism of action of BBR and other alkaloids, including quercetin, against the enzyme UDP-N-acetylpyruvate reductase (MurB). In the case of the pathogen *S.* Typhimurium, BBR has been shown to be an efficient inhibitor of the enzyme (in silico) and thus significantly reduced the ability of bacteria to synthesize peptidoglycan [172].

Cui et al., in their study, showed that the alkaloid BBR exhibited promising pharmacological interactions with colistin (COL) and EDTA in overcoming resistance to COL in *S. enterica* (10 MDR strains). Importantly, each substance individually was not particularly active against COL-resistant *S. enterica*; in contrast, the combination of COL with BBR or with BBR + EDTA significantly reduced the value of COL’s MIC (in the first case, by 8–2048 times, and in the second, by 128–131072 times, with BBR and EDTA concentration equal to ½ MIC). Importantly, BBR (½ MIC) also lowered the MIC for a number of other antibiotics, including doxycycline (DOX), florfenicol, CYP, and olaquindox. The checkerboard assay showed that the combination of BBR + EDTA + COL showed synergistic activity against all COL-resistant *Salmonella* strains, including the *mcr-1* (+) strain. Molecular analyses also showed that BBR interferes with *Salmonella* virulence factors, AcrAB-TolC and MCR-1, through direct binding to efflux pumps (docking analysis). Moreover, in combination with EDTA, the alkaloid disrupted the expression of the *mcr-1* and *tol*C genes. In addition, several changes were noted at the cellular level: increased perforation of the outer cell membrane due to restored COL sensitivity, accumulation of ROS, a decrease in ATP synthesis due to mitochondrial damage, and Krebs cycle disorder [173]. Wong et al. evaluated BBR activity against *S.* Typhimurium ATCC 14028. In the microdilution test, it was shown that the alkaloid was not significantly active against the pathogen, with a MIC of ~1000 mM. In the conducted test of the sensitization of the pathogen to ampicillin, no beneficial effect on the MIC of the antibiotic against *Salmonella* was shown (>2 times increase in the MIC of ampicillin) [174]. BBR isolated from *Berberis aristata* DC showed moderate activity against *S.* Typhi Ty(2). The MIC value was determined at ~50 µg/mL, and the Kirby–Bauer test showed a ZOI diameter of 15.5 mm vs. a CYP ZOI = 22.5 mm [175]. The activity of several alkaloids against the FtsZ cell division protein in *S.* Enteritidis was also evaluated. BBR hydrochloride was active against two *Salmonella* strains: CVCC3377 and ATCC14028 (MIC/MBC 0.32/0.64 mg/mL and >64 mg/mL, respectively). With regard to the FtsZ protein, BBR at a concentration of 4 µg/mL was shown to inhibit the activity of said GTPase by ~30%. During molecular analysis, a concentration-dependent manner of inhibition of the polymerization process via the FtsZ protein was demonstrated [176].

The cited literature presents a significant range in terms of the MIC value of berberine against various strains of *Salmonella* spp. Due to the uniform methodology of most studies (the microdilution broth method), this phenomenon should be seen in the differences between the tested strains. The topic requires in-depth and especially comparative research, including simultaneous testing of an alkaloid against various *Salmonella* strains.

*Shigella* spp.

*Rhizoma coptidis* extract has been studied for its antibacterial properties targeted at *S. dysenteriae*. In the analysis of UPLC and Q-TOF MS, it was shown that one of the main components was BBR and epi-BBR. Thermokinetic analysis showed that the BBR alkaloid was characterized by high bacteriostatic activity against the pathogen (54.10% inhibition of growth rate) [177]. The BBR isolated from the stems of *Berberis aristata* DC was also examined by Saumya et al. in relation to a range of food-borne pathogens. The MIC values for *S. sonnei*, *S. flexneri*, *S. boydii*, and *S. dysenteriae* were determined to be 50–100 μg/mL, depending on the strain. The most sensitive to BBR was *S. sonnei* NK 840 (MIC ~50 µg/mL). The Kirby–Bauer test compared BBR (100 µg/mL) activity with CYP (same concentration) activity against *Shigella* isolates. The ZOI values were obtained in the range of 9–19.5 mm for the tested species. In the case of the above-mentioned *S. sonnei* strain, the alkaloid exhibited comparable activity to CYP (ZOI = 19.5 mm vs. 25 mm) [175].

*Campylobacter* spp.

A few reports of BBR estimated activity against *Campylobacter* spp. come from the Fukamachi et al. study. Researchers evaluated the activity of Hangeshashinto extract on 29 different pathogens, including *C. jejuni* ATCC 29428. The MIC = MBC = 10 mg/mL values were demonstrated in the microdilution test. BBR was one of the 15 potential active ingredients of the extract; hence, it is difficult to assess how much it really contributed to the antibacterial effect of the preparation [178].

The summary of pharmacological interactions between alkaloids and antimicrobial preparations is presented in Table 2.

## 4. Sanguinarine

### 4.1. Characteristics

**Figure 5:**
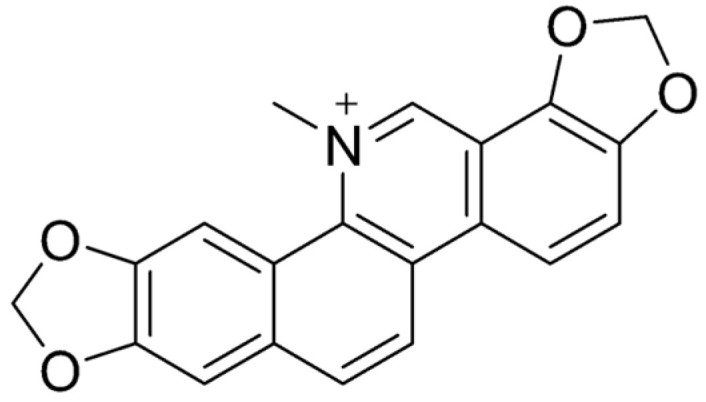
Molecular structure of SAN.

Sanguinarine (SAN) (Figure 5) is an orange-red benzophenanthridine alkaloid that is most commonly found as a chloride salt, though it can also occur as a sulfate salt. It has limited solubility in water but dissolves easily in organic solvents [179]. This crystalline compound is primarily extracted from plants belonging to the *Papaveraceae* family [180]. SAN is known for its wide range of biological effects, including anti-inflammatory [181,182], antimicrobial [183], antifungal [184], and antitumor properties [185]. In addition to these activities, SAN has shown significant potential in treating gastrointestinal disorders. For example, it has been effective in healing inflammatory lesions in the small intestine caused by non-steroidal anti-inflammatory drugs (NSAIDs), such as indomethacin [186]. It has also demonstrated therapeutic benefits in UC, where it helps improve colon length, reduce weight loss, and lower levels of pro-inflammatory cytokines [187]. Beyond its gastrointestinal applications, SAN’s potent antimicrobial properties make it a promising candidate for developing new antibiotics, particularly against challenging biofilm-forming bacteria like *Staphylococcus aureus* [188]. These diverse properties highlight its potential as a valuable compound for both medical and pharmaceutical research.

### 4.2. Impact on Intestinal Mucosa

SAN is widely known for its impact on intestinal and colon cells. SAN pretreatment using the IEC-6 cell (Intestinal Epithelial Cell 6; epithelioid cells of rat small intestine) enhanced cell viability and reduced apoptosis [189]. This suggests that SAN can protect intestinal epithelial cells from IR-induced damage. SAN was also found to suppress the activation of the HMGB1/TLR4 pathway, which is associated with inflammatory responses. This suppression was accompanied by a decrease in the levels of pro-inflammatory cytokines (IL-6, IL-8, TNF-α, and INF-γ) and an increase in the anti-inflammatory cytokine IL-10, further protecting intestinal tissues from inflammatory damage following radiation exposure. The protective effect of SAN was also evaluated against indomethacin-induced small bowel inflammatory lesions in male Sprague Dawley rats by Lin et al. [186]. Indomethacin caused significant weight loss, intestinal damage, and an inflammatory response in the rats. SAN, when administered, reversed these effects, demonstrating a dose-dependent protective effect. Specifically, SAN reduced weight loss, relieved pathological changes in the intestine, and improved the integrity of the small intestinal mucosal barrier [186]. It also decreased levels of tissue lactate dehydrogenase and improved the expression of tight junction proteins, especially ZO-1, which are crucial for maintaining the mucosal barrier. Additionally, SAN inhibited the inflammatory response and oxidative stress induced by indomethacin, reducing the levels of inflammatory markers like TNF-α, IL-6, and IL-1β, and improving antioxidant markers like SOD while reducing MDA. Furthermore, in cellular studies with IEC-6 cells, SAN protected against indomethacin-induced damage by decreasing membrane permeability and promoting barrier function [186]. The SAN effect was also evaluated on DSS-induced UC in C57BL/6 mice [187]. SAN significantly improved colon length, reduced weight loss, alleviated symptoms, and mitigated pathological colon injury in DSS-induced mice. The compounds decreased pro-inflammatory cytokines (TNF-α, IFN-γ, IL-1β, IL-6, IL-13, and IL-18) and increased anti-inflammatory cytokines (IL-4 and IL-10). It also suppressed the expression of NLRP3, caspase-1, and IL-1β. SAN inhibited NLRP3 inflammasome activation and caspase-1 activation in lipopolysaccharide-stimulated THP-1 cells at non-cytotoxic doses, similar to the effects of MCC950, a known NLRP3 inhibitor. Overall, SAN demonstrated significant potential in reducing inflammation and improving gut health in UC models. Another study involving the SAN effect on acetic acid induced this disease in Kunming mice [181]. Mice treated with SAN showed significant improvement in body weight and reduced macroscopic colonic damage compared to untreated colitis mice. SAN treatment significantly reduced histological signs of cell damage in the colon, such as necrosis, inflammation, and crypt distortion, indicating the start of tissue repair. The study found that SAN significantly reduced the expression of the p65-NF-κB protein, a marker associated with inflammation, in a dose-dependent manner. SAN reduced MPO activity, which is associated with neutrophil infiltration and acute inflammation, indicating a potential mechanism for its protective effects. Concurrently, the levels of pro-inflammatory cytokines such as TNF-α and IL-6 in both serum and colonic tissues were decreased by SAN, suggesting its anti-inflammatory properties. Overall, SAN demonstrated significant anti-inflammatory effects in the acetic acid-induced UC in the Kunming mice model, which is comparable to the effects in the DSS-induced UC in C57BL/6 mice [181,187].

**Figure 6:**
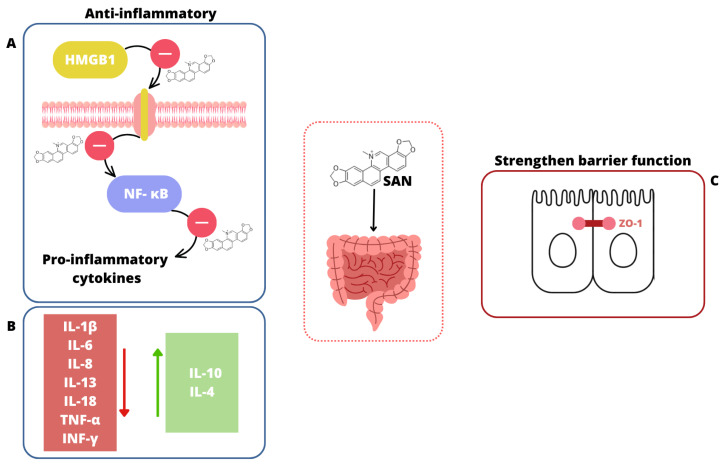
The impact of SAN on the intestines. (**A**) SAN suppresses the activation of the HMGB1/TLR4 pathway, reduces NF-κB expression, and lowers levels of pro-inflammatory cytokines [181,189]. (**B**) SAN decreases the level of pro-inflammatory cytokines (red square 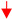) and increases the level of anti-inflammatory cytokines (green square 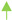) [181,187,189]. (**C**) SAN strengthens the intestinal barrier function through improving the expression of tight junction proteins, especially ZO-1 [186].

Overall, SAN shows promising protective effects against induced intestinal/colon damage, as presented in Figure 6.

### 4.3. Impact on Gut Microbiome and Its Clinical Implications

The impact of SAN on the human gut microbiota is summarized in Table 1, while other studies focused on animals. Gu et al. investigated the beneficial effects of SAN on intestinal injury induced by ionizing radiation (IR) [189]. IR caused dysbiosis in intestinal microbiota, i.e., increased the relative abundance of *Proteobacteria* and decreased that of *Firmicutes*, whereas pretreatment with SAN restored these changes [189]. *Proteobacteria* overrepresentation is associated with increased intestinal permeability and inflammation, as many *Proteobacteria* possess lipopolysaccharides (LPSs) in their outer membranes. The abundance of *Firmicutes* species enhances the gut’s mechanical barrier and supports immune homeostasis [190]. The abundance of *Lactobacillus* species was significantly increased in SAN-treated mice compared to the control group. *Lactobacillus* is known for its beneficial effects on gut health, including its role in enhancing the gut barrier and modulating the immune response. There was a notable decrease in the abundance of *Escherichia* species, which are often associated with inflammation and gut dysbiosis. Overall, whole abdominal irradiation-induced (WAI) alterations in 20 genera were rescued by SAN pretreatment, and 12 genera had opposite changes between WAI and SAN + WAI group [189]. The reduction in these potentially pathogenic bacteria suggests that SAN may contribute to a healthier gut environment by suppressing bacteria that could exacerbate inflammation or other adverse conditions following radiation exposure. SAN treatment resulted in a more balanced gut microbiota composition, characterized by a higher ratio of beneficial to pathogenic bacteria, which is critical for maintaining intestinal health and recovery post-radiation [191].

A similar experiment was performed by X. Li et al., who investigated the therapeutic effect of SAN on UC in a mouse model (C57BL/6) induced by dextran sulfate sodium (DSS) [187]. The analysis showed that in the UC model, there were significant differences in the relative abundance of five phyla, including *Proteobacteria*, *Bacteroidetes*, *Epsilonbacteraeota*, *Deferribacteres*, *Verrucomicrobia*, *Actinobacteria*, and *Tenericutes*, between the model group and a control group [187]. SAN treatment at a dose of 10 mg/kg reversed these changes. Specifically, SAN restored the balance of bacterial genera by increasing beneficial bacteria like *Muribaculaceae* and *Ruminiclostridium*_5 and decreasing pathogens such as *Escherichia-Shigella* [187]. This indicates that SAN can effectively improve intestinal microbiota imbalances in UC mice.

### 4.4. Antimicrobial Properties Against Various Enteroinvasive Bacterial Pathogens

*Listeria monocytogenes*

The Kudera et al. study confirmed that SAN is highly active against *L. monocytogenes* ATCC 7644. Importantly, the bacterial MIC of the alkaloids (equal to 16 μg/mL) was comparable to that of two important eradicators of this pathogen: CYP (MIC = 4 µg/mL) and VAN (MIC = 8 µg/mL) [171]. In the Osei-Owusu et al. study, SAN presented a very promising synergistic interaction with TET against *L. monocytogenes* ATCC 7644. Alkaloids were separately characterized by activity at the level of MIC = 3.33 μg/mL; in the subinhibitory concentration of 0.125 µg/mL, the preparation lowered the MIC for TET from 0.5 to 0.125 µg/mL, which resulted in an impressive synergistic effect. Significantly, at a concentration of 1 µg/mL, SAN led to a decrease in MIC for TET of 8-fold (from 0.5 to 0.06 µg/mL) [192]. The synergistic nature of the interaction between SAN and CYP against *L. monocytogenes* ATCC 7644 has also been demonstrated. The preparation showed activity against the pathogen by separately reaching MIC at 8 μg/mL, in combination with CYP alkaloids in the subinhibitory concentration of 2 µg/mL, which lowered the MIC for antibiotics by 104-fold (from 1.667 to 0.016 µg/mL), demonstrating strong synergistic action [193].

The distribution of the results is still unclear, especially since the alkaloid was tested against the same reference strain using the microdilution broth method. Extensive studies are required to evaluate the diversity of bacteria’s susceptibility to alkaloid drugs.

*Salmonella* spp.

Fan et al. have shown that SAN is also characterized by antibacterial activity against *S.* Enteritidis. Two strains of the pathogen, CVCC 3377 and ATCC 14028, were more susceptible to the alkaloid than to BBR and MIC/MBC, respectively, with values of 0.04/0.16 mg/mL and 0.16/0.32 mg/mL. However, in the field of FtsZ GTPase inhibition, SAN was less active than the above-mentioned alkaloid (an inhibition rate of less than 20%) [176]. Moreover, SAN was also moderately active against two clinically relevant *S.* Enteritidis and *S*. Typhimurium. MIC values for both pathogens were as follows: 256 and 512 μg/mL [171]. Zhu et al. evaluated the activity of 2,3-dihydro-1H-imidazo [2,1-a]isoquinolin-4-ium derivatives of SAN against *S.* Typhi CMCC(B)50071. The microdilution study showed that the derivatives and SAN exhibited moderate activity against the mentioned pathogen (MIC for derivatives: 128–256 µg/mL; MIC for SAN: 128 µg/mL) [194]. Zhang et al. demonstrated that the addition of a chlorine atom to the SAN molecule could enhance its inhibitory properties against the type III secretion system (T3SS) in *S.* Typhimurium, making it a promising compound. The study observed that SAN chloride inhibited the translation of a SipA–lactamase fusion protein in HeLa cells. Notably, at a concentration of 5 µM, SAN reduced the pathogen’s invasiveness to less than 5%, confirming its potential as a T3SS inhibitor. Additionally, SAN suppressed the expression of multiple genes within *Salmonella* pathogenicity island 1 (SPI-1) by interfering with the HilA transcription factor (a member of the OmpR/ToxR family). This disruption led to altered gene expression for *sip*A and *sip*B [59].

*Shigella* spp.

Kudera et al. also reported on the activity of SAN against the clinically relevant pathogen *S. flexneri*. The compound exhibited antibacterial activity, with a MIC of 64 μg/mL [171]. Another study evaluated the pharmacological interactions between SAN and TET against *S. flexneri*. Alkaloids were characterized by activity against the pathogen at the level of MIC = 16 µg/mL. No synergistic interaction was observed between the preparation and the antibiotic [192]. Osei-Owusu et al. also evaluated interactions between SAN and CYP—an antibiotic commonly used against food-borne pathogens. The study showed that although the alkaloid did not have a significant synergistic effect with CYP against *S. flexneri*, it was highly active against the pathogen (MIC= 16 μg/mL) [193].

*Yersinia enterocolitica*

A few reports on the activity of the alkaloid SAN against *Yersinia* spp. come from the study by Kudera et al. The researchers reported that the preparation showed a MIC for *Y. enterocolitica* at a level of 256 μg/mL [171]. As with *S. flexneri*, the mentioned alkaloid showed no synergistic interaction with TET against *Y. enterocolitica*. However, the preparation was characterized by a distinct activity against the pathogen, with a MIC value at the level of 64 µg/mL [192].

The summary of pharmacological interactions between alkaloids and antimicrobial preparations is presented in Table 3.

## 5. Chelerythrine

### 5.1. Characteristics

**Figure 7:**
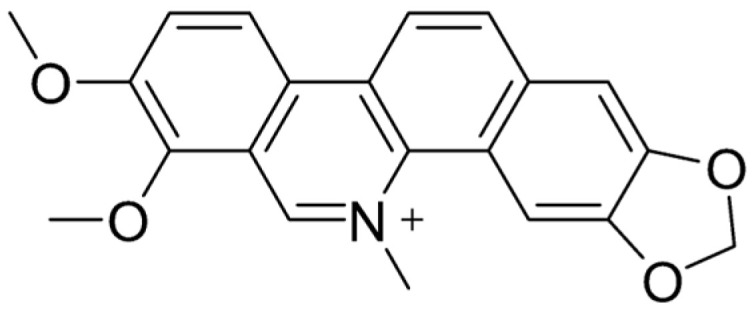
Molecular structure of CHE.

Chelerythrine (CHE) (Figure 7) is a yellow benzophenanthridine alkaloid [195,196] found in several plant families, including *Fumariaceae*, *Papaveraceae*, *Ranunculaceae*, and *Rutaceae* [197]. This compound is recognized for its diverse pharmacological properties, such as antitumor [198], antimicrobial [199], and anti-inflammatory effects [200]. Despite its potential, CHE’s poor solubility in water limits its bioavailability, a challenge shared by many drug candidates [154]. Research into its effects on the gastrointestinal (GI) tract has revealed promising therapeutic applications. For instance, CHE has demonstrated protective properties against ethanol-induced gastric ulcers in mice [200]. Additionally, it has been investigated for its role in promoting intestinal recovery following ischemia–reperfusion (IR) injury [117]. Beyond its GI benefits, CHE has shown significant potential as an antimicrobial and antibiofilm agent, particularly in addressing difficult-to-treat biofilm-associated infections. Its ability to act through multiple mechanisms, as well as its compatibility with combination therapies, makes it a strong candidate for further clinical explorations and developments [201,202].

### 5.2. Impact on Intestinal Mucosa

CHE is a nonselective protein kinase C (PKC) antagonist/inhibitor [203]. CHE was shown to significantly inhibit the chloride secretion induced by the *Aeromonas* spp. strain Sb supernatant [204]. The study reported that in the presence of CHE, the short-circuit current (ISC), which is indicative of chloride ion movement, was reduced from 42 ± 2 µA/cm^2^ to 32 ± 2 µA/cm^2^ (*p* < 0.005), indicating that PKC plays a role in the chloride secretion pathway activated by the *Aeromonas* β-hemolysin [204]. This could become a helpful intervention during gastrointestinal infections, of which diarrhea is a significant problem and is the third cause of death in children up to 59 months. Interestingly, CHE did not significantly alter the decrease in transepithelial resistance (Rt), which is another measure of epithelial barrier function [204]. This implies that while PKC is involved in the secretory response, it may not be directly responsible for the observed changes in epithelial barrier function (e.g., paracellular permeability) induced by *Aeromonas* β-hemolysin [204].

Chelerythrine chloride, as a PKC inhibitor, also reduces the activation of ezrin (ERM protein) and the apical localization of MRP2 in intestinal epithelial cells during *S.* Typhimurium infection [205]. Specifically, when cells were treated with CHE chloride prior to infection with this pathogen, there was a significant decrease in the movement of ezrin from the detergent-soluble to the detergent-insoluble (cytoskeletal) fraction, which is a marker of ezrin activation [205]. Since MRP2 is essential for the efflux of hepoxilin A3, a key chemoattractant that guides neutrophils to the site of infection, the reduction in MRP2 apical localization caused by CHE chloride consequently diminished neutrophil transmigration across the intestinal epithelium during infection [205]. This mechanism is essential for the pathogenesis of *Salmonella*, as it promotes neutrophil transmigration, a key feature of the inflammatory response during infection [205]. This implies that CHE chloride, through its inhibition of PKC, indirectly impairs a critical component of the host’s inflammatory response to the mentioned pathogen.

CHE demonstrated a protective effect against intestinal damage caused by ischemia–reperfusion injury by inhibiting apoptosis and maintaining intestinal structure, potentially through mechanisms independent of its PKC-inhibiting properties [117]. Western blot analysis revealed that CHE treatment reduced caspase-3 protein levels in IR rats, correlating with decreased apoptosis. While CHE did not significantly alter cell proliferation rates in IR rats, it did prevent the extensive apoptosis observed in these animals, thereby preserving enterocyte turnover. Moreover, CHE treatment led to increased mucosal weight, villus height, and crypt depth in the jejunum and ileum of IR rats compared to untreated IR animals [117]. These changes suggest improved structural integrity and potentially enhanced nutrient absorption due to increased surface area, indicating that CHE could be beneficial in therapeutic strategies aimed at mitigating intestinal IR injury.

### 5.3. Impact on Gut Microbiome and Its Clinical Implications

CHE influence on the human microbiota is complex. While it exerts antimicrobial effects that can inhibit pathogenic bacteria, many publications describe in vitro experiments measuring antibacterial properties by MIC, and its impact on commensal microbiota is less clear. Some studies suggest that CHE may selectively inhibit pathogenic bacteria without significantly disrupting beneficial microbial populations of farm animals [206,207,208,209]. This selective antimicrobial activity is crucial in maintaining a balanced microbiota, which is essential for overall intestinal health. While scientists mainly focus on the study of animal microbiota, the work of Li et al. highlights CHE (reducing butyrate synthesis by *Crostridiales* and protein enrichment from the *Proteobacteria phylum*). This study is important due to the influence of the natural compounds analyzed on the functional profiles of human gut microbiomes [210].

### 5.4. Antimicrobial Properties Against Various Enteroinvasive Bacterial Pathogens

The Tavares et al. study evaluated the activity of alkaloids isolated from *Zanthoxylum rhoifolium* as well as the antibacterial properties of their derivatives. CHE and its derivative, dihydro-chelerythrine (DCHE), were characterized by activity against several food-borne pathogens, including *S. sonnei* ATCC 25931 and *S.* Typhimurium ATCC 14028. For DCHEL, the following MIC/MBC values against bacteria were obtained: 100/>100 and 50/>100 µg/mL. Alkaloids had fourfold higher activity against the previously mentioned pathogens, with values of 25/100 and 25/>100 µg/mL, respectively [118]. CHE activity against *S.* Typhimurium SL1344 was also assessed by Wang et al. The researchers reported that the alkaloid achieved activity (MIC) comparable to oxacillin at ~256 µg/mL [157].

## 6. Overcoming Toxicity

The use of CHE and SAN as natural substances with antimicrobial and anticancer applications has many therapeutic benefits. However, the cytotoxicity of these compounds causes potentially negative effects not only on bacterial or cancer cells, but also on the cells of potential patients. In addition, poor bioavailability due to the hydrophobicity of these alkaloids, the first-pass effect (rapid metabolism in the gut and liver), low chemical stability, and pumping out of the enterocytes (P-glycoprotein activity) is also becoming a problem [211]. Scientists are constantly looking for solutions to these problems. Recent years of research have identified several possibilities for alternative modifications of the compound’s structure or forms of administration. The use of nanoparticles, modifications of the dihydro-cheleritrin/sanguinarine form, the attachment of additional atoms (e.g., chlorine) to the starting compounds, combinations with other drugs, or the use of extended-release forms could potentially mitigate adverse clinical effects [212].

So far, in this field, encapsulation of sanguinarine chloride (SC) with a sol–gel-based nanoparticle platform has been used as a potential treatment for melanoma. The combination led to an apoptosis of > 90% of B16 melanoma cells. Most importantly, the nanoparticle did not interact directly with the melanoma cell, while after just 2 h, the combination was as effective as the action of pure SAN after 24 h. This suggests that the use of nanoparticles leads to a regular and controlled release of the drug, providing the desired clinical benefit [213].

Liu et al.’s study also provided evidence that SC is a promising compound with anticancer activity. Indeed, it showed that it acts on the ROS/USP47/BACH1/HMOX1 axis and, through the induction of ferroptosis, effectively inhibits prostate cancer growth [214]. This regulated prostate cancer cell death and growth inhibition, highlighting the potential of sanguinarine derivatives as a therapeutic strategy for cancer treatment.

Highly significant findings were reported by Yan et al., demonstrating that SC exerts a potent suppressive effect on the expression of human telomerase reverse transcriptase (hTERT) and telomerase activity in cancer cells. This unique property of SC led to progressive telomere shortening and the inhibition of cellular proliferation. Ultimately, SC treatment triggered the activation of cellular senescence-associated pathways, including p16, p21, and p53, and resulted in the accumulation of senescence-associated β-galactosidase (SA-β-gal)-positive cells [215]. These results underscore the potential of SC in oncological applications.

The effects of CHE and SAN and their dihydro derivatives were evaluated on cultured hepatocytes, human lymphocytes and leukocytes, and rat peritoneal mast cells. Dose-dependent degranulation of mast cells was demonstrated only with CHE. Both alkaloids, on the other hand, inhibited leukocyte chemiluminescence, as well as E rosette formation, while having no effect on their derivatives [216].

Also, the concepts of combining different alkaloids seem to show promise for the future. Black salve, with one of its components being *Sanguinaria canadensis*, with the predominant presence of SAN and CHE, was tested against melanoma. Synergistic cytotoxicity of the alkaloids present against skin cancer cell lines was demonstrated, with IC50 values of 2.1 mM and IC50 values of 3.14 mM for A375 and A431 [217].

A number of studies report significant benefits of developing specific hybrid compounds composed of an alkaloid and molecules recognized by immune cells through PAMP/TLR interactions. Surface antigens of yeast fungi (including β-glucan) as a factor facilitating the uptake of an alkaloid from the gastrointestinal tract by macrophages is a potential future direction of research in this area [218,219]. Combinations of BRB with lactoferrin (Lf) are also interesting options from the point of view of combating infections in terms of the etiology of intracellular bacteria. A study by Andima et al. showed that the alkaloids BRB and SAN, in combination with vancomycin or imipenem, provided in a coated form by Lf molecules, effectively eradicated *Staphylococcus aureus* and *Mycobacterium abscessus* inside macrophages [220]. The described mechanism, based on the double uptake of siderophore, both by host cells and intracellular pathogens, seems promising to increase the bioavailability of alkaloids and their therapeutic effectiveness.

Future efforts should prioritize advanced drug delivery systems, structural modifications, and rigorous in vivo evaluations to mitigate toxicity while enhancing targeted efficacy, paving the way for these alkaloids to be integrated into modern pharmacotherapy.

## 7. Conclusions

In summary, BBR, CHE, and SAN each demonstrate significant potential as therapeutic agents to combat various gastrointestinal infections, primarily by wielding direct antimicrobial action and modulating the host’s immune response (Table 1). BBR has demonstrated efficacy against a range of bacterial pathogens, including *L. monocytogenes* and *Salmonella* spp., by disrupting bacterial cell structures, inhibiting biofilm formation, and modulating the gut microbiota. CHE has also demonstrated promise by inhibiting bacterial growth, disrupting biofilms, and modulating the inflammatory response within the gut. SAN, another alkaloid of interest, has shown effectiveness in healing inflammatory lesions, managing ulcerative colitis symptoms, and exhibiting antimicrobial properties against biofilm-forming bacteria. SAN modulates the intestinal environment by protecting intestinal cells, suppressing inflammatory pathways, and aiding in the restoration of the gut microbiota balance.

These alkaloids collectively demonstrate a multifaceted approach to combating infections, addressing pathogens directly while also modulating the host’s response to infection. Notably, BBR’s influence on gut microbiota and its ability to restore intestinal homeostasis in conditions like ulcerative colitis and type 2 diabetes highlight its therapeutic versatility. Similarly, CHE’s capacity to inhibit chloride secretion and its potential utility in managing diarrhea-related gastrointestinal infections further underscore the therapeutic potential of these compounds. The ability of these compounds to target bacterial virulence factors, disrupt biofilms, modulate the intestinal environment, and mitigate inflammatory responses collectively positions them as promising candidates for the development of novel therapeutic strategies. To fully harness their potential, further research, particularly well-designed clinical trials, is crucial to elucidate their therapeutic potential and optimize their application in the treatment of gastrointestinal infections.

However, despite its promising pharmacodynamic profile, the therapeutic use of BBR is currently severely limited by its poor bioavailability after oral administration, resulting mainly from its low solubility, poor intestinal absorption, and extensive first-pass metabolism. These challenges underscore the urgent need for advanced formulation strategies—such as nanoencapsulation or cyclodextrin-based systems—to enhance bioavailability and systemic efficacy. Research on controlling its release also appears crucial. Moreover, a thorough assessment of safety, including pharmacokinetics, cytotoxicity, and potential drug interactions, is essential to ensure clinical applicability. Addressing these aspects will be crucial to the transition of BRB and related alkaloids from promising preclinical agents to clinically viable therapeutic agents.

## Figures and Tables

**Table 1:** Effects on the intestinal mucosa and gut microbiome of the compounds described in this review.

	Berberine	Chelerythrine	Sanguinarine
**Microbiome Protein Composition**	Enrichment of proteins from specific bacteria: *Verrucomicrobia*, *Proteobacteria* ^2^, the *Bacteroidetes* phylum, the *Akkermansia* genus, and the *Verrucomicrobia* phylum ^1^	Enrichment of proteins from the *Bacteroides* genus and the *Akkermansia* genus	
**Activation of Bacterial Defense Mechanisms**	Leads to the upregulation of proteins associated with microbial defense and stress responses. This indicates that BBR induces a protective or stress response in the gut microbiome.	Activates protective bacterial mechanisms. This is evidenced by the increase in proteins related to microbial defense and stress responses. These include proteins associated with the cell wall/membrane/envelope biogenesis and signal transduction mechanisms.	Triggers bacterial protective mechanisms, as indicated by the increased abundance of proteins involved in microbial defense and stress responses. This includes proteins related to cell wall/membrane/envelope biogenesis, signal transduction mechanisms, and other stress-related functions.

Explanations: ^1^ The *Verrucomicrobia* phylum is associated with beneficial effects like improved gut barrier function and metabolic health; ^2^ *Proteobacteria* includes many species that are associated with gut inflammation and other adverse effects when they become overly abundant.

**Table 2:** Berberine-mediated enhancement of classic antimicrobial drug efficiencies.

Berberine			
Antibiotic/Chemotherapeutic	Type of Interaction	Pathogen Name	References
cyprofloxacin	synergistic	*S. enterica* subsp. enterica serovar Gallinarum	[164]
colistin		MDR *S. enterica*	[173]
streptomycin	additive	*L. monocytogenes* CMCC 54004	[160]
		*S. enterica* subsp. enterica serovar Typhimurium SL1344	

**Table 3:** Sanguinarine-mediated enhancement of classic antimicrobial drug efficiencies.

Sanguinarine			
Antibiotic/Chemotherapeutic	Type of Interaction	Pathogen Name	References
tetracycline	synergistic	*L. monocytogenes*	[192]
cyprofloxacin			[193]

## Data Availability

Data are contained within the article.
